# Traditional Chinese medicine method of tonifying kidney for hypertension: Clinical evidence and molecular mechanisms

**DOI:** 10.3389/fcvm.2022.1038480

**Published:** 2022-11-16

**Authors:** Qingqing Wang, Jianguo Lin, Cheng Li, Mingshan Lin, Qing Zhang, Xiaoxiao Zhang, Kuiwu Yao

**Affiliations:** ^1^Guang’anmen Hospital, China Academy of Chinese Medical Sciences, Beijing, China; ^2^Tianjin University of Traditional Chinese Medicine, Tianjin, China; ^3^Eye Hospital China Academy of Chinese Medical Sciences, Beijing, China; ^4^Beijing University of Chinese Medicine, Beijing, China

**Keywords:** tonifying kidney, hypertension, meta-analysis, network pharmacology, data mining

## Abstract

Hypertension is the most common chronic disease. A large amount of evidence showed that traditional Chinese medicine (TCM) method of tonifying kidney (TK) combined with routine treatment is more effective and safer in the treatment of hypertension. This study integrated meta-analysis, data mining, and network pharmacology to explore the efficacy and potential mechanisms of TK in the treatment of hypertension. Meta-analysis was performed to explore the efficacy and safety of TK combined with routine treatment in the treatment of hypertension. Data mining was used to screen the core herbs of the TK. Network pharmacology was used to predict the antihypertensive mechanism of TK core herbs. A total of 18 studies with 2,024 patients were included in this study. Meta-analysis showed that TK combined with routine treatment was superior to routine treatment alone in lowering blood pressure (systolic and diastolic blood pressures), lowering blood lipids (total cholesterol, triglyceride, low-density lipoprotein cholesterol), improving vascular endothelial functions (nitric oxide, endothelin) and TCM symptoms (headache dizziness, soreness, and weakness of waist and knees). In addition, TK was safe and has no obvious adverse reactions. Data mining showed that the core herbs of TK were *Eucommia ulmoides* Oliv. (Duzhong), *Vitex negundo* L. (Huangjing), *Taxillus chinensis* (DC.) Danser (Sangjisheng), *Ligustrum lucidum* W.T.Aiton (Nuzhenzi), *Astragalus mongholicus* Bunge (Huangqi), *Rehmannia glutinosa* (Gaertn.) DC. (Shudihuang). Network pharmacology predicted that core herbs antihypertensive components were oleanolic acid, ursolic acid, and civetone, and the antihypertensive targets were NOS3, NOS2, MMP9, TNF, PTGS2, HMOX1. In addition, the antihypertensive targets were enriched in cGMP-PKG signaling pathway, calcium signaling pathway, aldosterone-regulated sodium reabsorption, HIF-1 signaling pathway. In conclusion, TK combined with routine treatment for hypertension is effective and safe. The mechanism of TK may be related to GMP-PKG signaling pathway, calcium signaling pathway, aldosterone-regulated sodium reabsorption. On the premise of syndrome differentiation and treatment, it is promising to treat hypertension with TK.

**Systematic review registration:**

[https://www.crd.york.ac.uk/prospero/], identifier [CRD42022358276].

## Introduction

Hypertension is the most common chronic disease. The 2012–2015 China Hypertension Survey showed that 23.2% (≈244.5 million) of Chinese adults had hypertension, and another 41.3% (≈435.3 million) had prehypertension. Almost half of the hypertensive population were aware of their hypertension, 40.7% were treated, and only 15.3% achieved blood pressure control (1, 2). Elevated blood pressure is to blame for around half of all vascular fatalities in China. Systolic blood pressure (SBP) rises by 10 mmHg, increasing the risk of ischemic heart disease by around 30% (3). Hypertension is usually treated with western medicine for a long time, but this is often accompanied by problems such as decreased patient compliance, increased social and economic burden, and increased risk of adverse reactions (4–6). In summary, hypertension is a major public health challenge in the world. It is necessary to improve the ability to prevent and treat hypertension.

Traditional Chinese medicine (TCM) has a history of more than 2,000 years and is widely used in China to treat various diseases and has achieved remarkable curative effects. Substantial evidence is that TCM therapy is more effective and safer for hypertension (7–10). Hypertension is a “vertigo” in TCM. According to the theory of TCM, hypertension is related to the liver and kidney, and the main syndrome type is Yin deficiency and Yang excess. At present, more and more scholars believe that kidney deficiency is the key pathology of hypertension, especially kidney deficiency is closely related to senile hypertension (11). Most patients with hypertension have a long course of the disease and are complicated with coronary heart disease, hyperlipidemia, diabetes, and other chronic diseases. These diseases are closely related to kidney deficiency syndrome in the later stage, often with dizziness, forgetfulness, fatigue, waist and knee soreness, and other symptoms. The tonifying kidney (TK) method (such as Liuwei Dihuang pills, Jingui Shenqi pills, and Qiju Dihuang pills) can effectively treat many symptoms of hypertension of kidney deficiency type (12–14).

In recent years, there have been a large number of randomized controlled trials (RCTs) of classical prescription, self-designed prescription, and Chinese patent medicine in the treatment of hypertension according to the method of TK. However, there is still a lack of systematic reviews on the safety and efficacy of these studies, and the molecular mechanism of TK antihypertensive methods is also unclear. Network pharmacology can clarify the mechanism of drugs at the molecular level, explain the complex relationship between drugs and organisms, and analyze the synergistic effect of multiple drugs, which plays an important role in the mechanism discussion of TCM (15, 16).

Therefore, this study aimed to systematically review and evaluate the efficacy and safety of TK in the treatment of hypertension, and to explore the mechanism of TK by combining data mining and network pharmacology.

## Materials and methods

### Meta-analysis

This study followed the Preferred Reporting Items for Systematic Reviews and Meta-Analyses (PRISMA) (17). The study protocol (CRD42022358276) was registered in the PROSPERO.^1^

#### Eligibility criteria

(1) Types of Studies: RCTs of TK combined with routine treatment (RT) for the treatment of hypertension.
(2) Types of Participants: Participants who meet the diagnostic criteria of essential hypertension. The diagnostic criteria can refer to the diagnostic criteria of the Chinese Guidelines for the prevention and treatment of Hypertension 2018 and its past versions (18). The gender, age, race, onset time, and onset years of the participants were not restricted.
(3) Types of Interventions: The experimental group received TK combined with RT. The control group received RT. The dosage, usage, and course of treatment were not restricted.
(4) Types of Outcome Measures: The outcome measures were: clinical efficacy rate, SBP, diastolic blood pressure (DBP), total cholesterol (TC), triglyceride (TG), high-density lipoprotein cholesterol (HDL-C), low-density lipoprotein cholesterol (LDL-C), nitric oxide (NO), endothelin (ET-1), and TCM symptom.

#### Exclusion criteria

Exclusion criteria were as follows: (1) Duplicate published studies; (2) Studies with incorrect or incomplete data; (3) Unable to extract data for research; (4) Review and study on animal experiments.

#### Literature search strategy

The literature related to the efficacy and safety of TK for hypertension was searched in six electronic databases, including the China National Knowledge Infrastructure (CNKI), Wanfang Database, VIP Information Database, Cochrane Central Register of Controlled Trials, EMBASE, and PubMed, from the beginning to August 2022. The following keywords were used as search strategy and modified according to different databases: “tonifying kidney” “reinforcing kidney” “dihuang” “kidney nourishing” “hypertension” and “high blood pressure.”

#### Data extraction

Two researchers (ML and QZ) independently extracted and summarized all relevant data from the original literature, including the investigator, publication year, sample size, age, gender, diagnostic criteria, intervention, duration of intervention, and outcome measures. Any disagreements regarding data extraction will be discussed and resolved with the third researcher (XZ). When necessary, literature details could be asked by email. NoteExpress Reference Management Software was used to organize literature and generate citations.

#### Risk of bias and quality assessment

Two researchers (ML and QZ) assessed the risk bias of the included studies by the Cochrane Risk of Bias Assessment Tool (19), which included 7 items (1) random sequence generation; (2) whether allocation concealment was implemented; (3) whether the investigators and subjects were blinded; (4) whether the outcome evaluators were blinded; (5) whether the outcome data were complete; (6) whether there was selective outcome reporting; (7) whether there were other sources of bias. The evaluation was based on 3 evaluation criteria: low risk, high risk, and unclear risk. Any disagreements regarding the risk of bias and quality assessment will be discussed and resolved with the third researcher (XZ).

#### Statistical analysis

The Stata 17.0 software (Stata Corp., College Station, TX, USA) was applied to perform the meta-analysis. Standardized mean difference (SMD) was utilized for continuous outcomes. Risk ratio (RR) was utilized for dichotomous outcomes. All of them were expressed with a 95% confidence interval (CI). Heterogeneity was tested using the Q test. If *I*^2^ ≤ 50%, the fixed-effects model was selected, and if *I*^2^ > 50%, the random-effects model was selected. The publication bias was estimated by Egger’s test and funnel plot. Results were considered statistically significant when *p* < 0.05.

### Data mining

#### Data extraction of prescription

Two researchers (ML and QZ) independently extracted the TK prescriptions involved in the trials. The names of the herbs were standardized according to the Chinese Pharmacopoeia (20). Any disagreements regarding data extraction will be discussed and resolved with the third researcher (XZ). Excel 2019 software was used to establish the database.

#### Core prescription extraction

Association analysis was performed using IBM SPSS Modeler 18.0 software. The core herb pairs and herb groups were identified using the *Apriori* algorithm. The parameters were set to support ≥ 20%, confidence ≥ 90%, and the maximum number of antecedents was 2. Subsequently, core herbs were selected for network pharmacology analysis.

### Network pharmacology

#### Data preparation

BATMAN-TCM^2^ (21) was used to collect the active components and targets of core herbs. The parameters were set as score>30 and *P*< 0.05. GeneCards^3^ (22) and DisGeNET^4^ (23) were used to collect the targets of hypertension. GeneCards parameters were set as relevance score ≥ 4. DisGeNET parameters were set as gene-disease association score ≥ 0.2.

#### Protein–protein interaction and enrichment analysis

The obtained targets were submitted to the STRING database^5^ (24), for protein--protein interaction (PPI) analysis. Meanwhile, the Metascape database^6^ (25) was used for enrichment analysis, which included Kyoto encyclopedia of genes and genomes (KEGG), biological process (BP), cellular components (CC), and molecular function (MF). In addition, Cytoscape 3.9.1 software (26) was used to construct the correlation network. The networks included compound-target network and PPI network. CytoHubba plug-in was used to screen key targets.

#### Molecular docking

The interaction between the target and compound was predicted by Pymol software, Autodock Vina (27), and LigPlot + (28). The crystal structure of the target was obtained through the PDB database.^7^ The structure of the compounds was obtained through the PubChem database.^8^

## Results

### Meta-analysis results

#### Eligible studies

**FIGURE 1:**
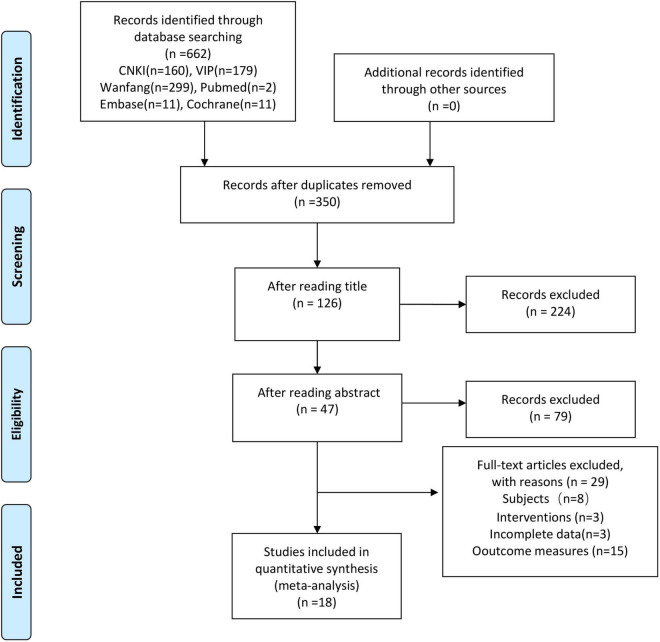
Flow diagram of the study screening.

A total of 662 studies were retrieved. After the elimination of duplications by NoteExpress and manual assistance, 350 studies remained. After reading the abstracts and titles, 47 studies remained. After reading the full text, 18 studies were finally included (29–46). The screening flow chart was shown in Figure 1.

#### Characteristics of studies

A total of 18 RCTs involving 2,024 patients (TK group: 1,014, RT group: 1,010) were included. All the studies were from China. The studies were published from 2013 to 2022. The longest intervention duration was 24 weeks, and the shortest was 4 weeks. The types of hypertension included essential hypertension, isolated systolic hypertension (ISH), and senile hypertension. The basic characteristics of all the included studies were shown in Table 1.

**TABLE 1:** Basic characteristics of included studies.

Study	Sample size (T/C)		Mean age (years) (T/C)		Intervention (T/C)	Duration (weeks)	Outcomes
Zhang and Li (29)	43	43	69.2 ± 3.6	69.7 ± 3.2	TK + RT/RT	8	①②③④
Huang and Xiao (30)	67	67	66.6 ± 4.8	66.2 ± 5.6	TK + RT/RT	8	①②⑤
Wang et al. (31)	75	75	70.2 ± 4.2	69.9 ± 4.4	TK + RT/RT	4	①②④⑤
Geng et al. (32)	41	41	71.44 ± 3.05		TK + RT/RT	4	②
Wang (33)	39	39	68.71 ± 2.86	69.24 ± 2.78	TK + RT/RT	12	①②
Sun et al. (34)	63	63	61.32 ± 5.71	60.74 ± 5.36	TK + RT/RT	12	①②
Guo (35)	60	60	61 ± 3.12	62 ± 1.17	TK + RT/RT	8	①②③④
Zhang (36)	45	45	69.94 ± 8.12	69.76 ± 8.01	TK + RT/RT	16	①②⑤
Ren et al. (37)	20	20	66.83 ± 2.35	67.93 ± 2.44	TK + RT/RT	8	②④
Teng et al. (38)	85	85	69.8 ± 6.1	69.2 ± 5.9	TK + RT/RT	12	①②④
Wu et al. (39)	65	65	71.28 ± 9.04	71.76 ± 8.28	TK + RT/RT	8	①②③
Zhu (40)	43	43	70.8 ± 3.7	74.8 ± 5.1	TK + RT/RT	24	②
Tian (41)	75	75	63.7 ± 5.3	64.1 ± 4.9	TK + RT/RT	8	②⑤
Chen and Liu (42)	90	90	72.6 ± 5.6	71.9 ± 5.8	TK + RT/RT	8	①②
Jiang et al. (43)	35	30	66.23 ± 5.02	66.35 ± 5.86	TK + RT/RT	12	①②
Wu et al. (44)	45	45	49.93 ± 3.49	48.34 ± 4.25	TK + RT/RT	8	②④
Li et al. (45)	66	66	67.4 ± 4.32	66.9 ± 4.3	TK + RT/RT	4	①③④
Lu et al. (46)	57	58	67.98 ± 11.05	68.78 ± 10.26	TK + RT/RT	24	②

T, treatment group (TK group); C, control group (RT group); TK, tonifying kidney treatment; RT, routine treatment; ① clinical efficacy rate; ② blood pressure; ③ blood lipids; ④ vascular endothelial function; ⑤ TCM syndromes.

#### Risk of bias

**FIGURE 2:**
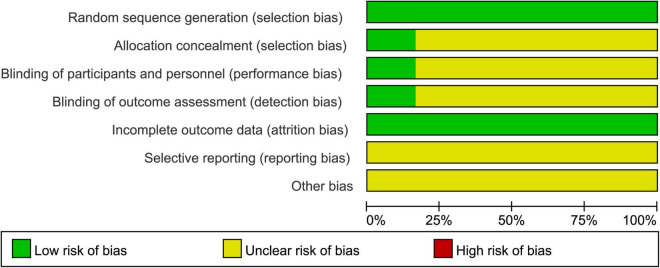
Risk of bias of included studies.

The results of the risk of bias were presented in Figure 2. All studies described the generation of random sequences (random number table). Three studies described allocation concealment and blinded (43, 44, 46). The remaining fifteen studies did not mention allocation concealment, blinded. Overall, the quality grade of the literature is not high.

#### Meta-analysis results

The calculated results of all meta-analyses were shown in Table 2.

**TABLE 2:** Calculation results of the meta-analysis.

Outcomes	Trials	Sample size	SMD/RR	95% CI	*Z*	*P*	*I*^2^ (%)	*P* for heterogeneity
Clinical efficacy rate	12	1,451	1.21	(1.16, 1.27)	8.39	0.00	0.00	0.94
**Blood pressure**								
HTN SBP	10	1,152	–0.95	(−1.32, −0.57)	–4.94	0.00	88.92	0.00
ISH SBP	7	740	–1.43	(−2.01, −0.85)	–4.83	0.00	91.89	0.00
HTN DBP	10	1,152	–0.98	(−1.37, −0.59)	–4.91	0.00	89.69	0.00
ISH DBP	7	740	0.06	(−0.29, 0.41)	0.35	0.72	81.89	0.00
**Blood lipids**								
TC	4	468	–1.47	(−2.34, −0.60)	–3.32	0.00	94.26	0.00
TG	4	468	–2.68	(−4.42, −1.15)	–3.43	0.02	97.49	0.00
LDL-C	4	468	–1.18	(−1.89, −0.46)	–3.22	0.00	92.21	0.00
HDL-C	4	468	0.17	(−0.48, 0.82)	0.51	0.61	91.87	0.00
**Endothelial function**								
NO	7	788	2.30	(0.73, 3.88)	2.86	0.00	98.75	0.00
ET-1	7	788	–1.66	(−2.42, −0.89)	–4.25	0.00	95.30	0.00
**TCM symptom**								
Headache dizziness	4	524	–3.24	(−5.84, −0.64)	–2.44	0.01	99.11	0.00
Soreness and weakness of waist and knees	4	524	–2.30	(−4.33, −0.27)	–2.22	0.03	98.93	0.00

#### Clinical efficacy rate

**FIGURE 3:**
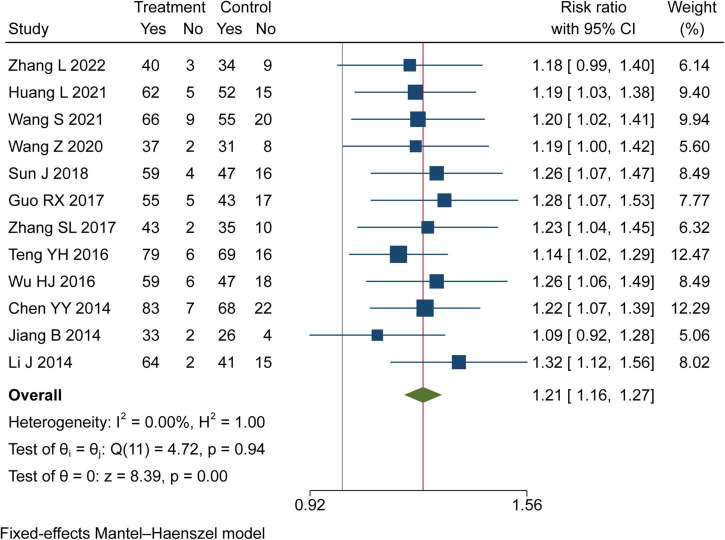
Forest plot of clinical efficacy rate.

Twelve trials assessed the clinical efficacy rate of TK on hypertension in this study. There were 733 patients in the TK group and 718 in the RT group. Meta-analysis indicated that TK combined with RT can significantly increase the clinical effective rate than RT (RR = 1.21, 95% CI [1.16, 1.27], *I*^2^ = 0%, *P*< 0.05, Figure 3).

#### Blood pressure

**FIGURE 4:**
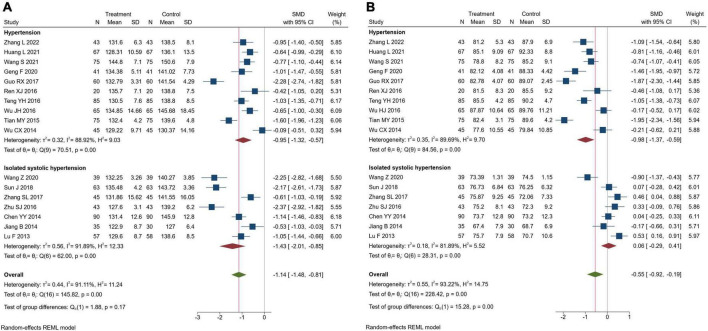
Forest plot of blood pressure. **(A)** Systolic blood pressure; **(B)** diastolic blood pressure.

Seventeen trials assessed blood pressure. There were 948 patients in the TK group and 944 in the RT group. Meta-analysis indicated that TK combined with RT could significantly reduce systolic and diastolic blood pressure compared with RT alone (SBP: SMD = −1.14, 95% CI [-1.48, -0.81], *I*^2^ = 91.11%, *P*< 0.05; DBP: SMD = −0.55, 95% CI [-0.92, -0.19], *I*^2^ = 93.22%, *P*< 0.05). Further, subgroup analysis was performed according to types of hypertension. In hypertensive patients, the subgroup analysis revealed that TK combined with RT could significantly reduce SBP and DBP compared with RT alone (SBP: SMD = −0.95, 95% CI [-1.32, -0.57], *I*^2^ = 88.92%, *P*< 0.05; DBP: SMD = −0.98, 95% CI[-1.37, -0.59], *I*^2^ = 89.69%, *P*< 0.05). In ISH patients, subgroup analysis revealed that TK combined with RT could significantly reduce SBP compared with RT alone (SMD = −1.43, 95% CI [-2.01, -0.85], *I*^2^ = 91.89%, *P*< 0.05), but there was no significant difference between RT and TK groups in reducing DBP (SMD = 0.06, 95% CI [-0.29, 0.41], *I*^2^ = 81.89%, *P* = 0.72), as shown in Figure 4. We performed a sensitivity analysis using the method of excluding studies one by one according to the Galbraith plot (Supplementary Figure 1), and the results showed that the heterogeneity was still more than 60%, but it did not affect the results, indicating that the results of the meta-analysis were reliable. We speculate that increased heterogeneity resulted from the use of the post-intervention mean. We once considered using mean changes for meta-analysis, but the correlation coefficient we obtained was less than 0.5, according to the Cochrane Handbook (Version 6.3, 6.5.2.8) (47), if a value less than 0.5 is obtained, then there is little benefit in using change from baseline.

#### Blood lipids

**FIGURE 5:**
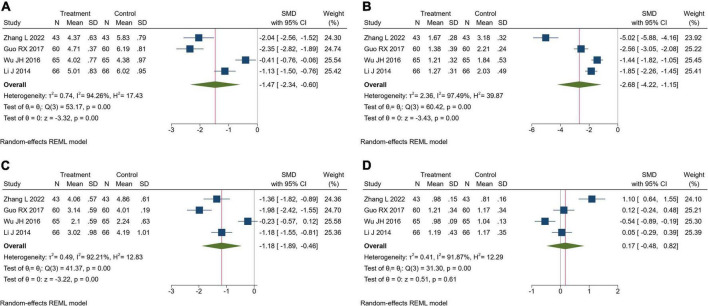
Forest plot of blood lipids. **(A)** Total cholesterol; **(B)** triglyceride; **(C)** low-density lipoprotein cholesterol; **(D)** high-density lipoprotein cholesterol.

Four trials assessed the blood lipids. There were 234 patients in the TK group and 234 in the RT group. Meta-analysis indicated that compared to the RT group, TK combined with RT could significantly improve TC (SMD = −1.47, 95% CI [-2.34, -0.60], *I*^2^ = 94.26%, *P*< 0.05, Figure 5A), TG (SMD = −2.68, 95% CI [-4.22, -1.15], *I*^2^ = 97.49%, *P*< 0.05, Figure 5B), LDL-C (SMD = −1.18, 95% CI [-1.89, -0.46], *I*^2^ = 92.21%, *P*< 0.05, Figure 5C), but there was no significant difference in HDL-C (SMD = 0.17, 95% CI [-0.48, 0.82], *I*^2^ = 91.87%, *P* = 0.61, Figure 5D). We speculate that the reason for the high heterogeneity is the use of post-intervention averages.

#### Endothelial function

**FIGURE 6:**
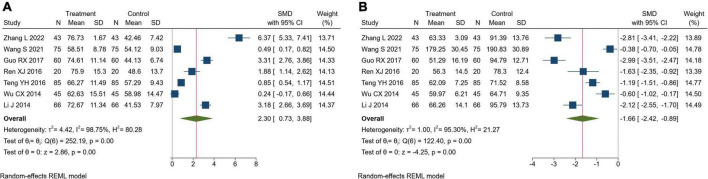
Forest plot of endothelial function. **(A)** Nitric oxide; **(B)** endothelin.

Seven trials reported endothelial function. There were 394 patients in the TK group and 394 in the RT group. Meta-analysis showed that TK combined with RT could significantly increase NO compared with RT alone (SMD = 2.30, 95% CI[0.73, 3.88], *I*^2^ = 98.75%, *P*< 0.05, Figure 6A). In addition, compared to the RT alone, TK combined with RT could significantly decrease ET-1 (SMD = −1.66, 95% CI [-2.42, -0.89], *I*^2^ = 95.30%, *P* < 0.05, Figure 6B). Sensitivity analysis suggested that the heterogeneity was caused by the different measurement methods and units of NO and ET-1.

#### Traditional Chinese medicine symptoms

**FIGURE 7:**
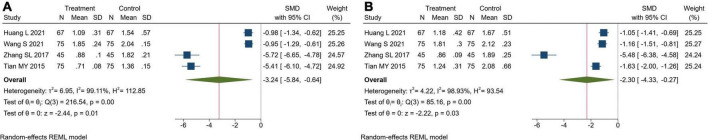
Forest plot of TCM symptoms. **(A)** Headache dizziness; **(B)** soreness and weakness of waist and knees.

Four trials reported TCM symptoms. There were 262 patients in the TK group and 262 in the RT group. Meta-analysis showed that TK combined with RT could significantly improve the TCM symptoms (Headache dizziness: SMD = −3.24, 95% CI [-5.84, -0.64], *I*^2^ = 99.11%, *P* = 0.01, Figure 7A; Soreness and weakness of waist and knees: SMD = −2.30, 95% CI [−4.33, −0.27], *I*^2^ = 98.93%, *P* = 0.03, Figure 7B). Sensitivity analysis showed that different evaluation criteria of the TCM scale resulted in large differences in scores, which may be the cause of heterogeneity.

#### Adverse reactions

Seven trials reported the safety of the drugs in this study (7/18, 38.89%) (31, 35, 37, 39, 42–44). Three trials no adverse reactions during the study (39, 42, 43). There were 5 cases of adverse reactions in the RT group and TK group in all trials, respectively (31, 35, 37, 44). Adverse reactions included dizziness, nausea, abdominal distension, diarrhea, skin flushing, etc. The symptoms of adverse reactions are mild, tolerable, and self-remitting. Serious adverse reactions and liver and kidney function damage occurred in all the trials.

#### Publication bias

**FIGURE 8:**
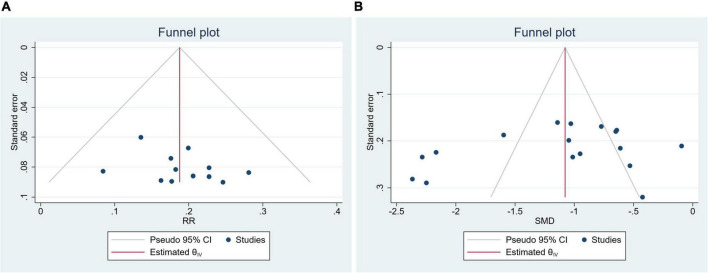
Funnel plot. **(A)** Clinical efficacy rate; **(B)** systolic blood pressure.

Clinical efficacy rate and SBP were evaluated by publication bias. The funnel plot and Egger’s test indicated no publication bias (Clinical efficacy rate: *P* = 0.2573, Figure 8A; SBP: *P* = 0.4199, Figure 8B).

### Data mining results

#### Characteristics of prescriptions

The basic characteristics of prescriptions were shown in Supplementary Table 1. A total of 18 prescriptions were included. There are 53 kinds of herbs in total. The most frequent occurrence of herbs was *Eucommia ulmoides* Oliv. (Duzhong, DZ), *Taxillus chinensis* (DC.) Danser (Sangjisheng, SJS), *Astragalus mongholicus* Bunge (Huangqi, HQ), *Rehmannia glutinosa* (Gaertn.) DC. (Shudihuang, SDH), *Achyranthes bidentata* Blume (Niuxi, NX). The functions of these herbs were mainly tonifying kidney and promoting blood circulation.

#### Herbs association rule analysis

**FIGURE 9:**
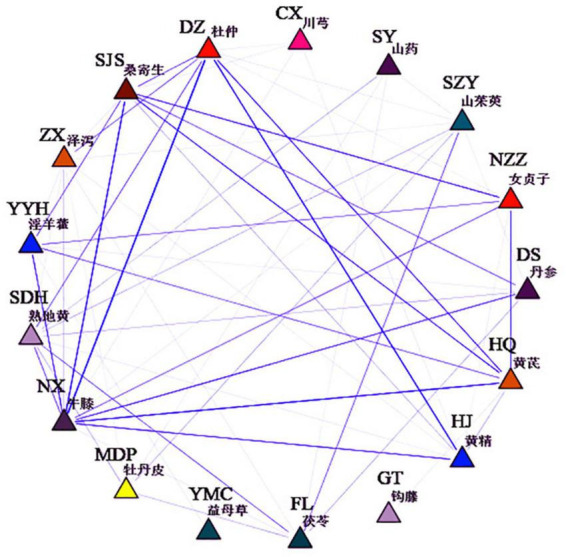
Herbs association network.

SPSS Modeler 18 was used to analyze the association rules of prescriptions, and the results were shown in Supplementary Table 2. The herbs association network was shown in Figure 9. Combined with the ranking of support degree and confidence degree, it can be seen that the core herb pairs were Duzhong-Huangjing, Huangqi-Nuzhenzi, Sangjisheng-Nuzhenzi, and the core herb group were Sangjisheng-Nuzhenzi-Huangqi, Niuxi-Nuzhenzi-Yinyanghuo, and Duzhong-Zexie-Niuxi. Based on the results of the data mining study, and also based on the principles of TK formulation, we selected Duzhong, Huangjing, Sangjisheng, Nuzhenzi, Huangqi, and Shudihuang as the core herbs for the network pharmacology study.

### Network pharmacology

#### Active compounds analysis

**FIGURE 10:**
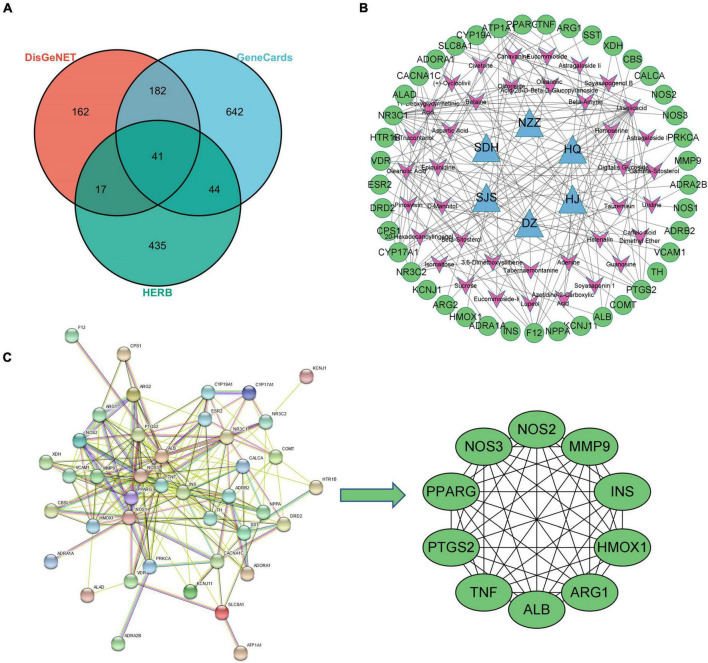
Network pharmacological analysis. **(A)** VEEN of disease and herb targets; **(B)** compound-target network; **(C)** screening of PPI key targets.

A total of 66 active ingredients and 537 targets corresponding to the active ingredients were collected. A total of 223 disease intersection targets were obtained. A total of 41 overlapping targets were obtained (Figure 10A). Subsequently, the compound-target network was made by Cytoscape (Figure 10B). Finally, analyzing network plug-in was used to screen the active compounds according to degree (Table 3).

**TABLE 3:** Key active compounds.

Degree	Name
22	Ursolic acid
16	Oleanolic acid
8	Civetone
8	11-Deoxyglycyrrhetinic acid
6	Pinosylvin
6	Tabernaemontanine
6	Gamma-Sitosterol
6	Oleanolic acid-28-O-Beta-D-Glucopyranoside
6	Canavanine
5	Betaine

#### Protein–protein interaction and gene enrichment analysis

**FIGURE 11:**
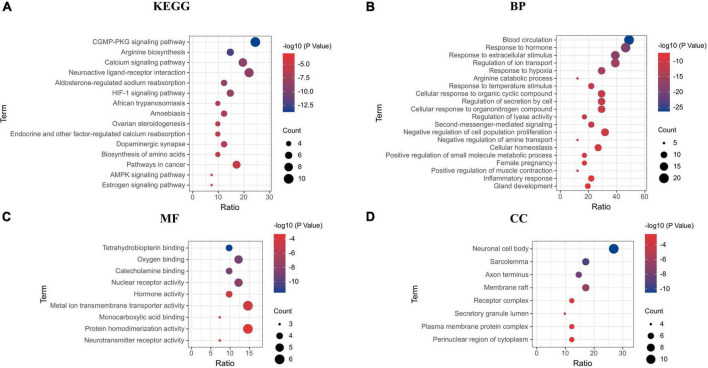
Gene enrichment analysis. **(A)** KEGG; **(B)** biological process; **(C)** molecular function; **(D)** cellular components.

STRING was used for PPI analysis, and then CytoHubba was used to screen key targets according to degree (Figure 10C) and using Cytoscape to screen key targets. The key targets were NOS3, NOS2, ALB, MMP9, TNF, PTGS2, ARG1, HMOX1, PPARG, and INS. Gene enrichment analysis indicated that the main KEGG items were cGMP-PKG signaling pathway, calcium signaling pathway, aldosterone-regulated sodium reabsorption, HIF-1 signaling pathway, endocrine and other factor-regulated calcium reabsorption, AMPK signaling pathway (Figure 11A); The main BP items were blood circulation, response to hormone, positive regulation of small molecule metabolic process, inflammatory response, cellular response to organonitrogen compound (Figure 11B); The main MF items were tetrahydrobiopterin binding, oxygen binding, catecholamine binding, monocarboxylic acid binding, neurotransmitter receptor activity (Figure 11C); The main CC items were neuronal cell body, sarcolemma, axon terminus, plasma membrane protein complex, perinuclear region of cytoplasm (Figure 11D).

#### Analysis of molecular docking

**FIGURE 12:**
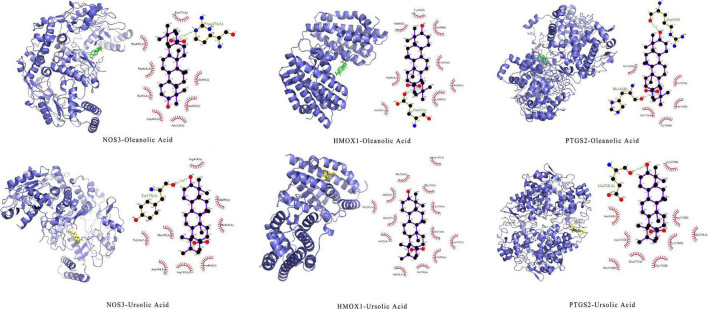
Molecular docking.

Through integrating data from sections “Active compounds analysis” and “Protein–protein interaction and gene enrichment analysis,” selected NOS3(PDBID:1m9j), HMOX1 (PDBID: 1n3u), PTGS2(PDBID: 5f19) as molecular docking proteins, selected oleanolic acid (CID: 10494), and ursolic acid (CID: 64945) as binding ligands. The results showed that the docking energy was ≤ −8 kcal⋅mol^–1^. Pymol and LigPlot + were used to draw the result of molecular docking (Figure 12).

## Discussion

Cardiovascular disease has become the disease with the highest morbidity and mortality in the world. Long-term hypertension is an important risk factor leading to the continuous increase in the prevalence of cardiovascular disease (48). The mechanism of hypertension is like an equation. The blood pressure equation can be approximated by Ohm’s law modified by fluid dynamics (pressure = flow × resistance), and the homeostasis system of blood pressure and its various hormonal regulators, including the vascular system, the central and sympathetic nervous systems, and the kidney, are the key to solving the hypertension equation (49). Fortunately, TCM is a great treasure trove, another key to unlocking the hypertension equation. According to TCM theory, “kidney governing water metabolism,” and the kidney has the function of regulating the metabolism of water and fluid in the human body. By warming the kidney Yang, the effect of water evaporation can be achieved. The elderly usually has kidney-jing deficiency, so tonifying the kidney can strengthen the body’s essence qi, activate immunity, and finally achieve the effect of lowering blood pressure.

This study was the first to integrate meta-analysis, data mining, and network pharmacology to explore the efficacy and potential mechanisms of TK in the treatment of hypertension. A total of 18 studies with 2,024 patients were included in this study. Meta-analysis showed that TK combined with RT was superior to RT alone in lowering blood pressure (SBP, DBP), lowering blood lipids (TG, TC, LDL-C), improving vascular endothelial functions (NO, ET-1) and TCM symptoms (headache dizziness, soreness and weakness of waist and knees). In addition, TK combined with RT was safe and has no obvious adverse reactions. It is worth noting that in patients with ISH, TK combined with RT did not differ from RT alone in lowering DBP. In this study, 88.8% (1,798 cases) of the subjects were senile hypertension, and 36.6% (740 cases) of the subjects were ISH. It can be speculated that TK is most suitable for elderly patients with kidney deficiency hypertension.

In this study, the data mining method was used to analyze and process the prescriptions involved in the TK. By analyzing the 18 prescriptions, we found that the TK used kidney-tonifying herbs as monarch herbs (such as Sangjisheng, Shudihuang, Duzhong, Nuzhenzi, Huangjing, Shanyao, Shanzhuyu), blood-activating and pulse-activating herbs as minister herbs (such as Danshen, Taoren, Honghua, Mudanpi, Chuanxiong), and water-disinhibiting herbs as adjuvant herbs (such as Fuling, Zexie, Yimucao). It is worth noting that most prescriptions were based on Liuwei Dihuang pills. Studies have shown that Liuwei Dihuang pills have the effect of regulating neurotransmitters in the brain, inhibiting the transformation of vascular smooth muscle cell phenotype, and improving immunity and anti-atherosclerosis (50–52). Next, through association analysis, we found that the core herb pairs of TK were Duzhong-Huangjing, Huangqi-Nuzhenzi, and Sangjisheng-Nuzhenzi. Finally, based on the theory of principle-method-recipe-medicines, we selected Duzhong, Huangjing, Sangjisheng, Nuzhenzi, Huangqi, and Shudihuang as the core herbs for the network pharmacology study.

The current principles of antihypertensive drugs include the excretion of natriuresis and diuresis, reducing blood volume; blocking calcium channels on the cell membrane of cardiomyocytes and vascular smooth muscle cells; and inhibiting the renin-angiotensin-aldosterone system. Thus, modulation of the balance between vasodilatory and vasoconstrictor targets is a potential measure of antihypertension. Through network pharmacology, we found that the main active components of TK were ursolic acid, oleanolic acid, civetone, pinosylvin, and the antihypertensive targets of TK were NOS3, NOS2, MMP9, TNF, PTGS2, HMOX1. Gene enrichment analysis showed that the antihypertensive targets were enriched in cGMP-PKG signaling pathway, calcium signaling pathway, aldosterone-regulated sodium reabsorption, HIF-1 signaling pathway, endocrine and other factor-regulated calcium reabsorption, and AMPK signaling pathway. The NO-cGMP signaling pathway is a well-known pathway for blood pressure regulation, and drugs directly targeting this pathway are currently the most promising new antihypertensive drugs (53). NO is a gas signaling molecule synthesized by three different isoforms of NOS enzymes: neuronal NOS (nNOS, NOS1), inducible NOS (iNOS, NOS2), and endothelial NOS (eNOS, NOS3). NO is produced in the vascular endothelial cells because it is fat-soluble, quickly permeates the cell membrane, and diffused downward into the smooth muscle cells, relaxing them, dilating blood vessels, and ultimately lowering blood pressure (54). Oleanolic acid is a widely distributed, bioactive pentacyclic triterpenoid. Studies have shown that oleanolic acid can prevent dexamethasone-induced hypertension in rats, which may be related to the antioxidant and NO release (55). Similarly, Bachhav et al. found that oleanolic acid reduced hypertension in L-NAME hypertensive rats by diuretic, and nephroprotective effects (56). Ursolic acid is a natural pentacyclic triterpenoid that activated both NO/cGMP and H_2_S/K_*ATP*_ pathways, resulting in synergistic vasodilation (57). In addition, Ursolic acid can reduce blood lipids and prevent atherosclerosis by regulating the balance of vasoactive components (ET-1, eNOS, Thromboxane A2, vascular cell adhesion molecule-1) (58). Finally, molecular docking was performed to further demonstrate the interaction between the active components and the targets. The results showed that the receptor and ligand were stably bound with stable hydrogen bonds.

This meta-analysis has limitations: Most of the included studies were Chinese clinical trials with small sample sizes, and the study protocol was not strictly implemented. Due to the lack of long-term follow-up of patients, it is not possible to confirm the long-term effect of integrated TK combined with RT in the treatment of hypertension. The methodological quality of the included studies was generally low, and the random sequence generation, random concealment scheme, and blinded implementation were not clearly stated. There were great differences in the variety, dosage, and course of treatment of the intervention measures. Among the outcome measures, TCM syndrome scores were quite subjective, statistical methods were quite different, and lack of a unity scale. It is hoped that in the future, TCM research can scientifically express the high-quality research evidence produced by the evidence-based practice of TCM in the internationally common “language.”

## Conclusion

In conclusion, this study showed that TK combined with RT for hypertension (clinical efficacy rate, SBP, DBP, TC, TG, LDL-C, NO, ET-1, headache dizziness, soreness and weakness of waist and knees) is effective and safe. The mechanism of TK may be related to GMP-PKG signaling pathway, calcium signaling pathway, aldosterone-regulated sodium reabsorption. On the premise of syndrome differentiation and treatment, it is promising to treat hypertension with tonifying kidney method.

## Data availability statement

The datasets used and/or analyzed during the current study are available from the corresponding author on reasonable request.

## Author contributions

QW and KY designed the manuscript. QW and JL edited the manuscript. ML, QZ, and XZ extracted and summarized the data. CL, JL, and QW revised the manuscript. All authors contributed to the manuscript revision, read, and approved the submitted version.

## Footnotes

1
https://www.crd.york.ac.uk/prospero/

2
http://bionet.ncpsb.org.cn/batman-tcm/

3
https://www.genecards.org/

4
https://www.disgenet.org

5
https://string-db.org/

6
https://metascape.org

7
https://www.rcsb.org/

8 https://pubchem.ncbi.nlm.nih.gov/compound/10494 and https://pubchem.ncbi.nlm.nih.gov/compound/64945
