# Supplementary material for: Traditional Chinese medicine method of tonifying kidney for hypertension: Clinical evidence and molecular mechanisms
Source: Front Cardiovasc Med. 2022 Nov 16;9:1038480. doi: 10.3389/fcvm.2022.1038480 (PMC9709460; doi:10.3389/fcvm.2022.1038480)
Supplement: Supplementary file 1 [file Data_Sheet_1.docx]

Supplementary Material


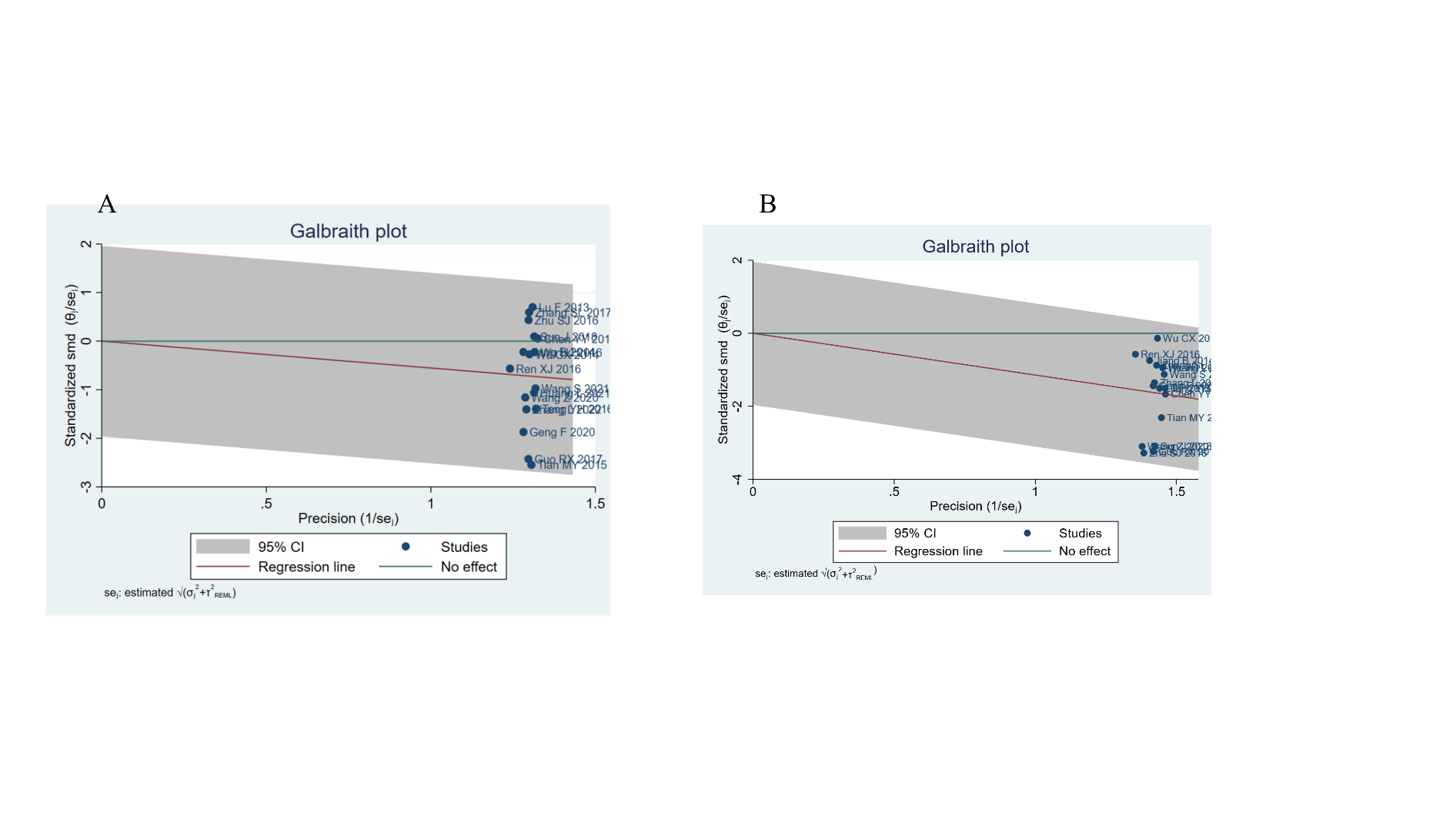


**Supplementary Figure 1.** Galbraith plot of blood pressure. (A) Diastolic blood pressure; (B) Systolic blood pressure.

**Supplementary Table 1. Basic characteristics of prescriptions**

| Study | Prescriptions | Components |
| --- | --- | --- |
| Zhang L 2022 | Bushen Huoxue Decoction | *Taxillus chinensis* (DC.) Danser (Sangjisheng, SJS) 15g, *Ligustrum lucidum* W.T.Aiton (Nuzhenzi, NZZ) 15g, *Uncaria rhynchophylla* (Miq.) Miq. (Gouteng, GT) 12g, *Leonurus japonicus* Houtt. (Yimucao, YMC) 15g, *Gastrodia elata* Blume (Tianma, TM) 12g, *Epimedium brevicornu* Maxim. (Yinyanghuo, YYH) 12g, *Achyranthes bidentata* Blume (Niuxi, NX) 15g, *Salvia miltiorrhiza* Bunge (Danshen, DS) 10g, *Astragalus mongholicus* Bunge (Huangqi, HQ) 9g |
| Huang L 2021 | Bushen Xifeng Decoction | *Eucommia ulmoides* Oliv. (Duzhong, DZ) 30g, *Cornus officinalis* Siebold & Zucc. (Shanzhuyu, SZY) 15g, *Concha Haliotidis* (Shijueming, SJM) 15g, *Uncaria rhynchophylla* (Miq.) Miq*.*(Gouteng, GT) 15g, *Reynoutria multiflora* (Thunb.) Moldenke (Yejiaoteng, YJT) 15g, *Rehmannia glutinosa* (Gaertn.) DC. (Shudihuang, SDH) 12g, *Astragalus mongholicus* Bunge (Huangqi, HQ) 12g, *Achyranthes bidentata* Blume (Niuxi, NX) 15g, *Poria* (Fushen, FS) 12g, *Ophiopogon japonicus* (Thunb.) Ker Gawl. (Maidong, MD) 10g, *Gastrodia elata* Blume (Tianma, TM) 10g, *Glycyrrhiza uralensis* Fisch. ex DC*.* (Zhigancao, ZGC) 10g, *Chrysanthemum* × *morifolium* (Ramat.) Hemsl. (Juhua, JH) 10g, *Platycladus orientalis* (L.) Franco (Baizhiren, BZR) 10g |
| Wang S 2021 | BushenYijingTiansui Decoction | *Cuscuta chinensis* Lam*.* (Tusizi, TSZ) 15g, *Rehmannia glutinosa* (Gaertn.) DC. (Shudihuang, SDH)15g, *Colla carapacis et plastri testudinis* (Guibanjiao, GBJ) 10g, *Cornus officinalis* Siebold & Zucc. (Shanzhuyu, SZY) 15g, *Lycium barbarum* L*.* (Gouqizi, GQZ) 20g, Gelatin of Buckhorn (Lujiaojiao, LJJ) 10g, *Dioscorea oppositifolia* L*.* (Shanyao, SY) 15g, *Achyranthes bidentata* Blume (Niuxi, NX)20g, *Platycladus orientalis* (L.) Franco (Baizhiren, BZR) 15g, *Eucommia ulmoides* Oliv. (Duzhong, DZ)15g, *Vitex negundo* L. (Huangjing, HJ) 15g, Albizia julibrissin *Durazz.* (Hehuanpi, HHP) 15g |
| Geng F 2020 | Bushen Huoxue Decoction | *Panax notoginseng* (Burkill) F.H.Cheng (Sanqi, SQ) 5g, *Paeonia* × *suffruticosa* Andrews (Mudanpi, MDP) 10g, *Alisma plantago-aquatica* L. (Zexie, ZX) 15g, *Chrysanthemum* × *morifolium (Ramat.)* Hemsl*.* (Juhua, JH) 15g, *Dioscorea oppositifolia* L*.* (Shanyao, SY) 15g, *Cornus officinalis* Siebold & Zucc*.* (Shanzhuyu, SZY) 15g, *Eucommia ulmoides* Oliv. (Duzhong, DZ) 15g, *Rehmannia glutinosa* (Gaertn.) DC. (Shudihuang, SDH) 30g, *Lycium barbarum* L*.* (Gouqizi, GQZ) 30g, *Poria* (Fuling, FL) 30g |
| Wang Z 2020 | Bushen Hemai Decoction | *Astragalus mongholicus* Bunge (Huangqi, HQ) 30g, *Eucommia ulmoides* Oliv. (Duzhong, DZ) 15g, *Taxillus chinensis* (DC.) Danser (Sangjisheng, SJS) 30g, *Vitex negundo* L. (Huangjing, HJ) 15g, *Ligustrum lucidum* W.T.Aiton (Nuzhenzi, NZZ) 30g, *Epimedium brevicornu* Maxim. (Yinyanghuo, YYH) 30g, *Achyranthes bidentata* Blume (Niuxi, NX) 15g, *Alisma plantago-aquatica* L. (Zexie, ZX) 30g, *Conioselinum anthriscoides ‘Chuanxiong’* (Chuanxiong, CX) 15g, *Angelica sinensis* (Oliv.) Diels (Danggui, DG) 15g, *Pheretima* (Dilong, DL) 9g |
| Sun J 2018 | Bushen Hemai Decoction | *Taxillus chinensis* (DC.) Danser (Sangjisheng, SJS) 10g, *Salvia miltiorrhiza* Bunge (Danshen, DS) 10g, *Rehmannia glutinosa* (Gaertn.) DC. (Shudihuang, SDH) 15g, *Cullen corylifolium* (L.) Medik. (Buguzi, BGZ) 15g, *Eucommia ulmoides* Oliv*.* (Duzhong, DZ) 15g, *Achyranthes bidentata* Blume (Niuxi, NX)15g, *Conioselinum anthriscoides ‘Chuanxiong’* (Chuanxiong, CX) 10g, *Vitex negundo* L*.* (Huangjing, HJ) 10g, *Acorus gramineus* Aiton (Shichangpu, SCP)10g, *Corydalis yanhusuo* (Y.H.Chou & Chun C.Hsu) W.T.Wang ex Z.Y.Su & C.Y.Wu (Yanhusuo, YHS) 10g |
| Guo RX 2017 | Bushen Huoxue Decoction | *Taxillus chinensis* (DC.) Danser (Sangjisheng, SJS) 15g, *Ligustrum lucidum* W.T.Aiton (Nuzhenzi, NZZ) 15g, *Epimedium brevicornu* Maxim. (Yinyanghuo, YYH)15g, *Astragalus mongholicus* Bunge (Huangqi, HQ) 10g, *Uncaria rhynchophylla* (Miq.) Miq.(Gouteng, GT) 19g, *Leonurus japonicus* Houtt.(Yimucao, YMC) 15g, *Salvia miltiorrhiza* Bunge (Danshen, DS) 15g, *Achyranthes bidentata* Blume (Niuxi, NX)15g, *Poria* (Fuling, FL) 15g |
| Zhang SL 2017 | Bushen Huazhuo Decoction | *Rehmannia glutinosa* (Gaertn.) DC. (Shudihuang, SDH) 12g, *Poria* (Fuling, FL) 10g, *Plantago asiatica* L. (Chenqianzi, CQZ) 9g, *Achyranthes bidentata* Blume (Niuxi, NX) 12g, *Thlaspi arvense L* (Baizhu, BZ)9g, *Thlaspi arvense* L*.* (Baijiangcao, BJC) 10g, *Salvia miltiorrhiza* Bunge (Danshen, DS) 9g, *Corydalis yanhusuo* (Y.H.Chou & Chun C.Hsu) W.T.Wang ex Z.Y.Su & C.Y.Wu (Yanhusuo, YHS) 9g, *Phellodendron amurense* Rupr. (Huangbo, HB)12g, *Nelumbo nucifera* Gaertn. (Lianzixin, LZX) 12g, *Acorus gramineus* Aiton (Shichangpu, SCP)10g, *Cullen corylifolium* (L.) Medik. (Buguzi, BGZ)12g, *Dioscorea collettii var. hypoglauca* (Palib.) S.J.Pei & C.T.Ting (Bixie, BX) 10g |
| Ren XJ 2016 | Bushen Hemai Decoction | *Taxillus chinensis* (DC.) Danser (Sangjisheng, SJS) 15g, *Astragalus mongholicus* Bunge (Huangqi, HQ) 30g, *Vitex negundo* L. (Huangjing, HJ) 15g, *Eucommia ulmoides* Oliv. (Duzhong, DZ) 15g, *Ligustrum lucidum* W.T.Aiton (Nuzhenzi, NZZ) 15g, *Epimedium brevicornu* Maxim. (Yinyanghuo, YYH) 30g |
| Teng YH 2016 | Bushen Huazhuo Decoction | *Rehmannia glutinosa* (Gaertn.) DC. (Shudihuang, SDH) 30g, *Lycium barbarum* L. (Gouqizi, GQZ) 20g, *Cornus officinalis* Siebold & Zucc. (Shanzhuyu, SZY)10g, *Alisma plantago-aquatica* L. (Zexie, ZX) 10g, *Poria* (Fuling, FL) 10g, *Paeonia* × *suffruticosa* Andrews (Mudanpi, MDP) 10g, *Taxillus chinensis* (DC.) Danser (Sangjisheng, SJS) 20g, *Eucommia ulmoides* Oliv. (Duzhong, DZ) 15g, *Apocynum venetum* L. (Luobuma, LBM) 15g, *Pinellia ternata* (Thunb.) Makino (Fabanxia, FBX)10g, *Citrus × aurantium* L. (Zhishi, ZS) 15g, *Prunus persica* (L.) Batsch (Taoren, TR) 10g, *Carthamus tinctorius* L. (Honghua, HH) 6g, *Achyranthes bidentata* Blume (Niuxi, NX) 15g, *Citrus × aurantium* L*.* (Zhiqiao, ZQ)10g, *Salvia miltiorrhiza* Bunge (Danshen, DS) 20g |
| Wu HJ 2016 | Bushen Dihuang Decoction | *Rehmannia glutinosa* (Gaertn.) DC. (Shudihuang, SDH) 15g, *Cornus officinalis* Siebold & Zucc*.* (Shanzhuyu, SZY) 15g, *Dioscorea oppositifolia* L*.* (Shanyao, SY) 15g, *Achyranthes bidentata* Blume (Niuxi, NX) 15g, *Alisma plantago-aquatica* L*.* (Zexie, ZX)9g, *Poria* (Fuling, FL) 9g, *Salvia miltiorrhiza* Bunge (Danshen, DS) 9g, *Paeonia* × *suffruticosa* Andrews (Mudanpi, MDP) 9g, *Eucommia ulmoides* Oliv. (Duzhong, DZ) 9g, *Reynoutria multiflora* (Thunb.) Moldenke (Yejiaoteng, YJT) 9g, *Vitex negundo* L*.* (Huangjing, HJ)15g, *Kigelia africana* (Lam.) Benth. (Cijili, CJL) 15g, *Panax notoginseng* (Burkill) F.H.Cheng (Sanqi, SQ) 6g, *Hairy Antler*(Lurong, LR) 6g |
| Zhu SJ 2016 | Bushen Hemai Decoction | *Astragalus mongholicus* Bunge (Huangqi, HQ), *Vitex negundo* L. (Huangjing, HJ), *Rehmannia glutinosa* (Gaertn.) DC*.* (Shudihuang, SDH), *Eucommia ulmoides* Oliv. (Duzhong, DZ), *Achyranthes bidentata* Blume (Niuxi, NX), *Lycium barbarum* L*.* (Gouqizi, GQZ) |
| Tian MY 2015 | Bushen Quyu Decoction | *Cornus officinalis* Siebold & Zucc. (Shanzhuyu, SZY) 12g, *Salvia miltiorrhiza* Bunge (Danshen, DS) 15g, *Paeonia* × *suffruticosa* Andrews (Mudanpi, MDP) 12g, *Taxillus chinensis* (DC.) Danser (Sangjisheng, SJS) 15g, *Poria* (Fuling, FL) 6g, *Acorus gramineus* Aiton (Shichangpu, SCP) 12g, *Carthamus tinctorius* L. (Honghua, HH) 6g, *Pheretima* (Dilong, DL) 10g, *Reynoutria multiflora* (Thunb.) Moldenke (Heshouwu) 12g |
| Chen YY 2014 | Bushen Huazhuo Decoction | *Rehmannia glutinosa* (Gaertn.) DC. (Shudihuang, SDH) 20g, *Rehmannia glutinosa* (Gaertn.) DC. (Shengdihuang, SDH) 20g, *Dioscorea oppositifolia* L. (Shanyao, SY) 20g, *Cornus officinalis* Siebold & Zucc. (Shanzhuyu, SZY) 20g, *Paeonia* × *suffruticosa* Andrews (Mudanpi, MDP) 12g, *Poria* (Fuling, FL) 30g, *Alisma plantago-aquatica* L. (Zexie, ZX)10g, *Cuscuta chinensis* Lam. (Tusizi, TSZ) 20g, *Uncaria rhynchophylla* (Miq.) Miq.(Gouteng, GT) 15g, *Leonurus japonicus* Houtt. (Yimucao, YMC) 15g, *Epimedium brevicornu* Maxim*.* (Yinyanghuo, YYH) 6g, *Angelica sinensis* (Oliv.) Diels (Danggui, DG)15g |
| Jiang B 2014 | Bushen Hemai Decoction | *Pheretima* (Dilong, DL) 10g, *Astragalus mongholicus* Bunge (Huangqi, HQ) 30g, *Vitex negundo* L*.* (Huangjing, HJ) 15g, *Epimedium brevicornu* Maxim. (Yinyanghuo, YYH) 30g, *Alisma plantago-aquatica* L*.* (Zexie, ZX)30g, *Angelica sinensis* (Oliv.) Diels (Danggui, DG) 15g, *Conioselinum anthriscoides ‘Chuanxiong’* (Chuanxiong, CX)12g, *Eucommia ulmoides* Oliv. (Duzhong, DZ) 15g, *Ligustrum lucidum* W.T.Aiton (Nuzhenzi, NZZ)15g, *Achyranthes bidentata* Blume (Niuxi, NX) 15g, *Taxillus chinensis* (DC.) Danser (Sangjisheng, SJS) 15g |
| Wu CX 2014 | Bushen Qinggan Granule | *Gastrodia elata* Blume (Tianma, TM) 30 g, *Uncaria rhynchophylla* (Miq.) Miq. (Gouteng, GT) 15g, *Eucommia ulmoides* Oliv. (Duzhong, DZ) 30 g, *Astragalus mongholicus* Bunge (Huangqi, HQ)15 g, Ilicis Cornutae Folium Immayuri (Kudingcha, KDC) 15g |
| Li J 2014 | Bushen Huoxue Decoction | *Ligustrum lucidum* W.T.Aiton (Nuzhenzi, NZZ) 15g, *Taxillus chinensis* (DC.) Danser (Sangjisheng, SJS)20, *Epimedium brevicornu* Maxim. (Yinyanghuo, YYH)15g, *Astragalus mongholicus* Bunge (Huangqi, HQ)10g, *Achyranthes bidentata* Blume (Niuxi, NX)15g, *Salvia miltiorrhiza* Bunge (Danshen, DS) 10g, *Leonurus japonicus* Houtt. (Yimucao, YMC) 15g |
| Lu F 2013 | Bushen Hemai Decoction | *Astragalus mongholicus* Bunge (Huangqi, HQ), *Vitex negundo* L. (Huangjing, HJ), *Taxillus chinensis* (DC.) Danser (Sangjisheng, SJS), *Epimedium brevicornu* Maxim. (Yinyanghuo, YYH), *Eucommia ulmoides* Oliv. (Duzhong, DZ), *Achyranthes bidentata* Blume (Niuxi, NX), *Alisma plantago-aquatica* L. (Zexie, ZX), *Conioselinum anthriscoides ‘Chuanxiong’* (Chuanxiong, CX), *Ligustrum lucidum* W.T.Aiton (Nuzhenzi, NZZ) |

**Supplementary Table 2**. Core herb pairs combinations in the results of association analysis.

| **Latter term** | **Former term** | **Support (%)** | **Confidence (%)** |
| --- | --- | --- | --- |
| Duzhong | Huangjing | 44.44444444 | 100 |
| Huangqi | Nuzhenzi | 38.88888889 | 100 |
| Sangjisheng | Nuzhenzi | 38.88888889 | 100 |
| Sangjisheng | Nuzhenzi and Huangqi | 38.88888889 | 100 |
| Huangqi | Nuzhenzi and Niuxi | 38.88888889 | 100 |
| Niuxi | Nuzhenzi and Yinyanghuo | 38.88888889 | 100 |
| Shanzhuyu | Mudanpi and Fuling | 27.77777778 | 100 |
| Shudihuang | Mudanpi and Shanzhuyu | 27.77777778 | 100 |
| Duzhong | Zexie and Niuxi | 27.77777778 | 100 |
| Niuxi | Chuanxiong and Huangjing | 22.22222222 | 100 |
